# Supplementary material for: Large-scale photonic chip based pulse interleaver for low-noise microwave generation
Source: Nat Commun. 2025 Jun 6;16:5252. doi: 10.1038/s41467-025-59794-z (PMC12144144; doi:10.1038/s41467-025-59794-z)
Supplement: Supplementary file 1 — Supplementary Information [file 41467_2025_59794_MOESM1_ESM.pdf]

Supplementary Information for:
Large-scale photonic chip based pulse interleaver for low-noise microwave generation

Zheru Qiu[1,2,#], Neetesh Singh[3,***,#], Yang Liu[1,2], Xinru Ji[1,2], Rui Ning Wang[1,2,5], Franz X. Kärtner[3,4,**], Tobias Kippenberg[1,2,*]
[1] *Swiss Federal Institute of Technology Lausanne (EPFL), CH-1015 Lausanne, Switzerland*
[2] *Center for Quantum Science and Engineering, EPFL, CH-1015 Lausanne, Switzerland*
[3] *Center for Free-Electron Laser Science, Deutsches Elektronen-Synchrotron, 22607 Hamburg, Germany*
[4] *Department of Physics, Universität Hamburg, Jungiusstr. 9, 20355 Hamburg, Germany*
[5] *Currently with Luxtelligence SA, CH-1015 Lausanne, Switzerland*
#: These authors contributed equally to this work.

Contents

1. Dispersion of the Si_3N_4 waveguides 1

2. Characterization of the time delays in the interleaver 2

3. Optical spectrum of input and interleaved pulse trains 3

4. Average photocurrent saturation at high optical power 4

5. Saturation behavior of a PIN photodetector 4

Supplementary Note 1. Dispersion of the Si_3N_4 waveguides

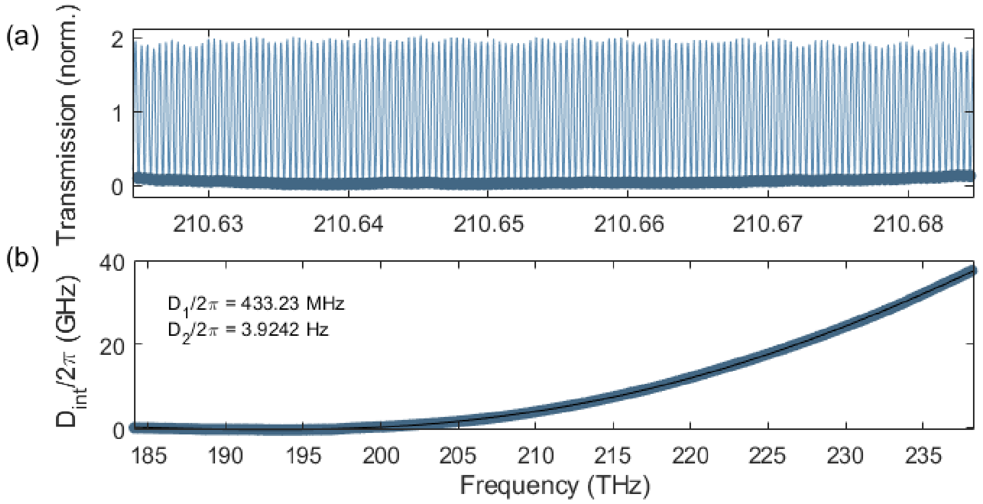

FIG. S1. **Dispersion characterization** (a) High resolution transmission spectrum of the single stage MZI test device with the local minima labeled. (b) The fitted integrated dispersion curve of the points in (a).

We characterized the dispersion of the delay lines by analysing the frequency-comb calibrated transmission spectrum of a single stage Mach-Zehnder interferometer interleaver (device number `D10602F03C06`). We located the local minima in the transmission spectrum and analysed the difference of their positions from a regular grid with a repetition rate

* tobias.kippenberg@epfl.ch
** franz.kaertner@desy.de
*** neetesh.singh@desy.de

of $D_1/(2\pi)$, which were then represented as the integrated dispersion D_{int} as shown in Figure S1.

$$D_1 = \frac{1}{2}(\omega_{+1} - \omega_{-1}) = \Delta\omega_{FSR} = \frac{2\pi c}{n_g L_R} = \frac{2\pi}{\beta_1 L_R} = 2\pi \times 433.23\,\text{MHz} \tag{1}$$

$$D_2 = \omega_{+1} - 2\omega_0 + \omega_{-1} = -\frac{4\pi^2\beta_2}{\beta_1^3 L_R^2} = -\frac{\beta_2 D_1^2}{\beta_1} = 2\pi \times 3.9242\,\text{Hz} \tag{2}$$

From the polynomial expansion coefficients D_i of the $D_{\text{int}}(\mu)$ as a function of the number μ of the local minima, we extracted the group index of the waveguide to be $n_g = 2.0977$ and the group velocity dispersion constant D to be $+18.3\,\text{ps}/(\text{nm}\cdot\text{km})$ (anomalous). The D is close to the $\sim +18.0\,\text{ps}/(\text{nm}\cdot\text{km})$ specified value of standard SMF-28 telecom fiber, which accumulates to 0.3 ps for the maximum propagation length and 30 nm bandwidth. The simulated dispersion parameters in the main text were obtained with a mode solver (Lumerical) using the Sellmeier fit on the experimentally measured refractive index of the Si_3N_4 film (Woollam).

Supplementary Note 2. Characterization of the time delays in the interleaver

We take advantage of the frequency domain response of the cascaded MZI interleaver to precisely determine the delay lengths. We perform high-resolution optical spectroscopy with a home-built optical vector network analyzer [1]. We can prove that the delay length of the longest stage in the cascaded MZI device can be characterized by the Fourier transform of the optical transmission spectrum, where the time delay is represented as the position of the highest frequency peak in the transformed domain.

The complex transfer function of a cascaded MZI interleaver with N stages can be modeled by the transfer matrix formalism:

$$\mathcal{C}_n = \begin{pmatrix} \sqrt{1-k_n} & i\sqrt{k_n} \\ i\sqrt{k_n} & \sqrt{1-k_n} \end{pmatrix}, \mathcal{P}_n = \begin{pmatrix} \mathrm{e}^{i\beta(f)L_n} & 0 \\ 0 & 1 \end{pmatrix} \tag{3}$$

$$\mathcal{T} = \prod_{n=1}^{N} \mathcal{C}_n \mathcal{P}_n \mathcal{C}_7 \tag{4}$$

$$\mathcal{T}_{11} = \sum_{k=0}^{2^N-1} a_k \exp\left(i\beta(f) \cdot \sum_n L_n s_{kn}\right) \tag{5}$$

,where the $\mathcal{C}_n$ is the transfer matrix of the tunable directional coupler with a coupling ratio of k, and the $\mathcal{P}_n$ is the phase shift experienced in the nth stage delay line of propagation constant $\beta(f) = nf/c$ and length L_n. Here we neglect the effect of dispersion in the analysis window. The transfer matrix $\mathcal{T}_n$ of the entire 4 port system is the product of the transfer matrices corresponding to each stage. The complex field transmission of a single port $\mathcal{T}_{11}$ to another can be written as (5) by collecting the terms with the same complex exponential, where a_k are real constants and the s_{kn} is the nth bit of the binary representation of integer k.

The transmitted intensity $T(f)$ is

$$T(f) = \mathcal{T}_{11}\mathcal{T}_{11}^* \tag{6}$$

$$= \sum_k \sum_{k'} a_k a_{k'} \exp\left(i\beta(f) \cdot \left(\sum_n^N L_n s_{kn} - \sum_n^N L_n s_{k'n}\right)\right) \tag{7}$$

Let $L_n = 2^{n-1}L_1 + \Delta L_n$, where the $\Delta L_n = c\Delta t_n/n_g$ is the error in the delay length. We can see the terms in T with the factor of $\exp(i\beta L_1 \cdot 2^{N-1})$ can only come from the $n = N$ case in the summation, as $\sum_{n=1}^{N-1} 2^{n-1} = 2^{N-1} - 1 < 2^{N-1}$, such that these terms will come together with the factor of $\exp(i\beta\Delta L_N)$. When performing a Fourier transform to $T(f)$, the highest "frequency" peak would be at $n_g(2^{N-1}L_1 + \Delta L)/c = 2^{N-1}n_g L_1/c + \Delta t_N$. The Δt_N is then derived as the offset between the peak in the "frequency" domain (or "delay time domain") spectrum and the demanded delay length by the mode-locked laser used in the experiment.

We extracted the delay lengths of the shorter stages by repeating the same characterization after disabling the longer stages with focused ion beam (FIB). The FIB cuts were performed on the spirals with an Xe ion beam (FEI Helios G4). The chip used in the experiment was from the same wafer of the characterized chip (Number `D10701`, Field 1 and 4). Multiple waveguides were simultaneously cut at an angle, ensuring a complete loss of transmission and a low back reflection.

Supplementary Note 3. Optical spectrum of input and interleaved pulse trains

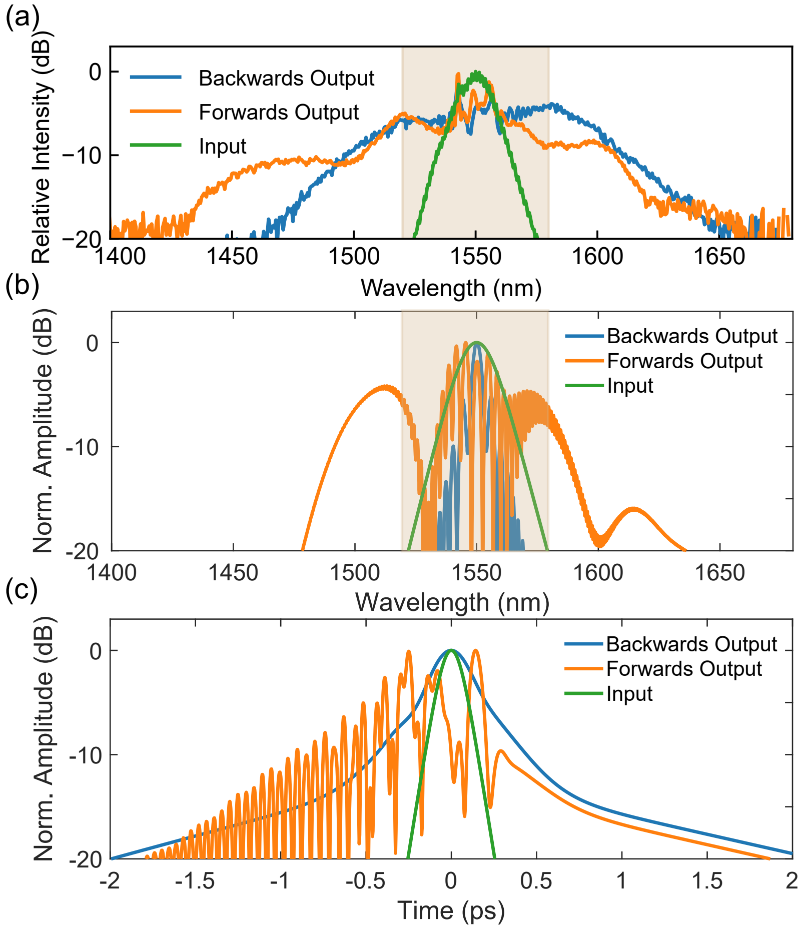

FIG. S2. **Nonlinear Spectrum Broadening** (a) Measured optical spectrum of the input pulse train and the output from the interleaver chip in backwards and forwards operation. (b) Output optical spectrum simulated by numerical solution of the nonlinear Schrödinger equation (NLSE). (c) The simulated output pulse shape in time domain.

Figure S2(a) shows the measured optical spectrum of the input pulse train from the mode-locked erbium fiber laser and the output from the interleaver chip. We tested the device by launching the pulse from the forward and backward direction. In the backward direction, the pulse goes through the short interleaver stages first. Whereas in the forward direction, the pulse starts from the long interleaver stages. In the red and orange traces, the pulse input is at the shortest stage (backwards) and the longest stage (forwards) of the interleaver, respectively. The coupled total output power from the chip was kept at ~ 3.1 mW. In both cases, the laser spectrum is broadened by the nonlinear processes in the Si_3N_4 delay lines. The broadening in the forwards case has distinctive sideband structures at ~ 1430 nm and ~ 1600 nm, which may indicate that there is a generation of incoherent supercontinuum.

Pulse propagation simulation by solving the NLSE in the forward and backward direction with the split-step Fourier method confirmed the spectrum broadening in the waveguides (Fig. S2(b)). In the simulation, We used the waveguide dispersion parameters of $\beta_2 = -2.09 \times 10^2 \mathrm{ps}^2/\mathrm{m}$, $\beta_3 = -5.07 \times 10^{-4} \mathrm{ps}^3/\mathrm{m}$ and a fixed launched power for both directions. Figure S2(c) shows that in the case of forwards operation, the pulses randomly break up in the time domain and disrupt the regular interval timing, while the pulses have retained the single Gaussian shape in backwards

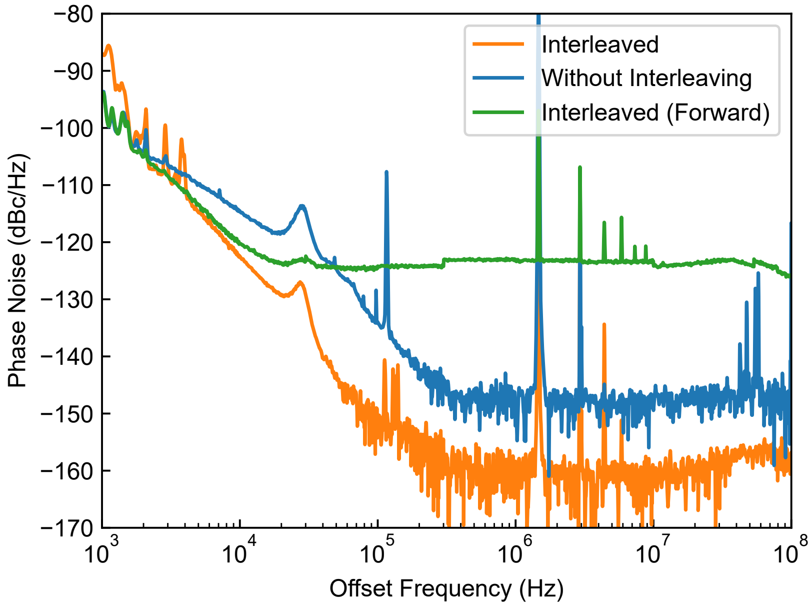

FIG. S3. Measured phase noise of the generated 13.87 GHz microwave, comparing forwards operation and backwards operation of the interleaver.

operation. As we can see in the forward direction the bandwidth of the spectrum is broader and the temporal shape of the pulse is more structured than in the backward direction. That is because of the stronger nonlinear effect experienced by the pulse in the forward direction compared to that in the backward direction. The pulse enters with relatively higher power into the long sections of the interleaver from the forward direction compared to the backward direction, that is because before the pulse reaches the longer sections of the interleavers from the backward direction it has been split multiple times by the directional couplers present at every stage. The higher power in the forward direction causes the pulse to experience higher nonlinear effects and thus shorter soliton fission length, as the fission length is inversely proportional to the peak power $(1/\sqrt{P})$ [2, 3].

Figure S3 shows the phase noise of generated microwave measured when the device is in forwards operation in comparison to the case of no interleaving and backwards operation. In forwards operation, the noise floor degraded to ~ -125 dBc/Hz due to the incoherent nonlinear process. In future work, to prevent modulation instability and pulse splitting, the waveguide GVD can be modified to normal by reducing the waveguide thickness or reducing the waveguide width.

Supplementary Note 4. Average photocurrent saturation at high optical power

We observed the average photocurrent also shows saturation at high input average power when the non-interleaved pulses are sent to the MUTC PD. Meanwhile, the interleaved pulses were not causing appreciable saturation for < 75 mW average power (Figure S4). The average photocurrent was measured from the bias port of the Freedom Photonics FP1015A, at the same 5.3 V bias voltage as in the microwave generation experiment.

Supplementary Note 5. Saturation behavior of a PIN photodetector

PIN photodetectors are of lower cost than the MUTC photodetectors but are known to suffer more from saturation when illuminated with short pulses. We observed a similar saturation of 64th harmonic power when the pulse is not interleaved when a Thorlabs DXM30AF photodetector is used for microwave generation. As shown in Fig. S5, the generated 13.9 GHz power saturated at -37 dBm. For the interleaved pulse train from the chip, no power dependent saturation was observed up to -27 dBm generated power at 2.6 mW optical power. The EDFA was not used in this experiment, which limited the average optical power and the microwave power in the interleaved case. As no power dependent saturation was observed, we expect the advantage of interleaved pulses to be larger than 11 dB if the setup was optimized for higher output power or the pulses can be amplified. We note that although the Thorlabs DXM30AF

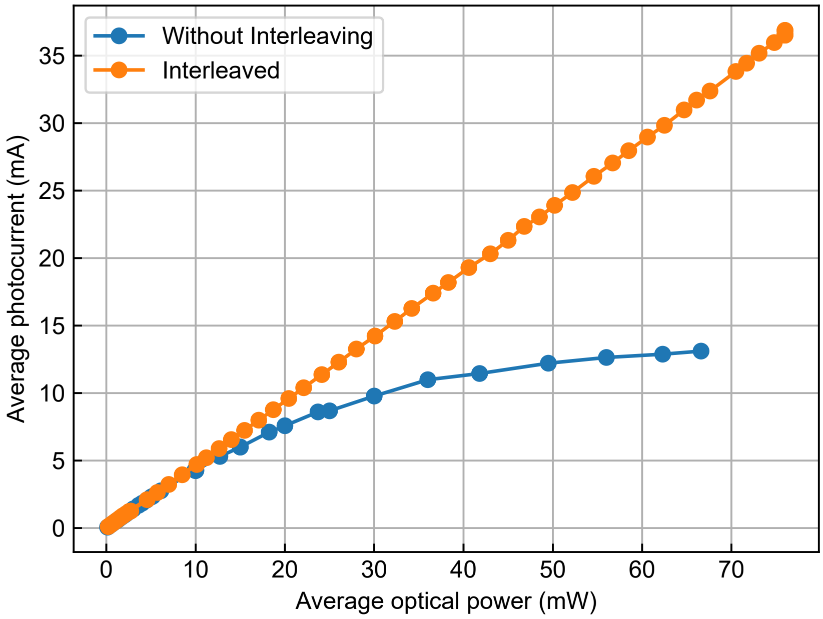

FIG. S4. Average photocurrent as a function of average input power at the MUTC photodetector.

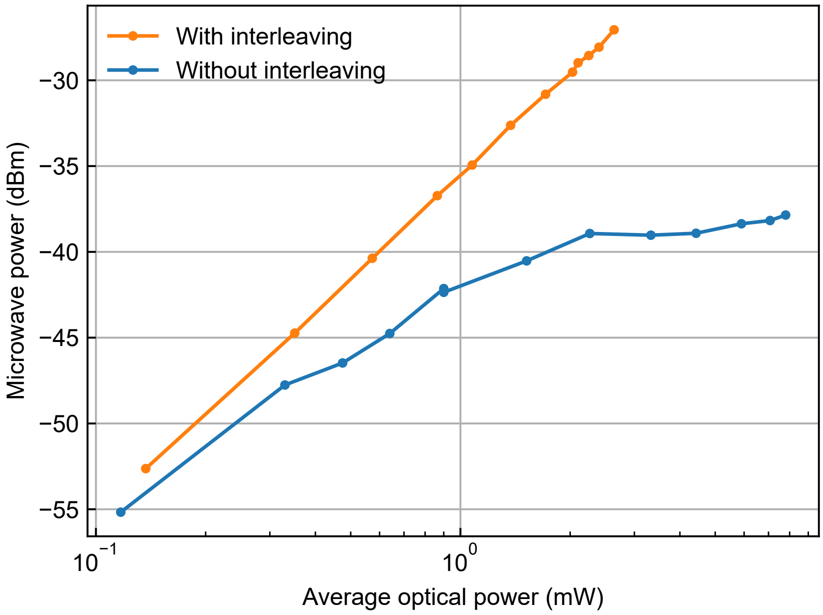

FIG. S5. Generated 13.9 GHz microwave power with the Thorlabs DXM30AF photodiode as a function of the input average optical power.

is specified with a higher responsivity (0.75 A/W at 1550 nm) than the Freedom photonics FP1015A MUTC PD (0.6 A/W at 1550 nm), the generated microwave power is 11 dB lower than the MUTC detector at 2.6 mW average power of interleaved pulses. This indicates the PIN photodetector may have a stronger power-independent saturation when illuminated with short pulses.

Supplementary References

[1] J. Riemensberger, N. Kuznetsov, J. Liu, J. He, R. N. Wang, and T. J. Kippenberg, Nature **612**, 56 (2022).

[2] J. M. Dudley, G. Genty, and S. Coen, Rev. Mod. Phys. **78**, 1135 (2006).
[3] N. Singh, D. Vermulen, A. Ruocco, N. Li, E. Ippen, F. X. Kärtner, and M. R. Watts, Opt. Express **27**, 31698 (2019).
